# Supplementary material for: Cytoprotective Compounds in the Primate Eye: Baseline Metabolomic Profiles of Macaca fascicularis Ocular Tissues
Source: Int J Mol Sci. 2025 Nov 7;26(22):10816. doi: 10.3390/ijms262210816 (PMC12652150; doi:10.3390/ijms262210816)
Supplement: Supplementary file 1 [file ijms-26-10816-s001.zip › ijms-3945115-supplementary.pdf]

Supplementary Information

for

Cytoprotective Compounds in the Primate Eye: Baseline Metabolomic Profiles of Macaca fascicularis Ocular Tissues

Maxim V. Fomenko [1], Lyudmila V. Yanshole [1], Vadim V. Yanshole [1], Elena Y. Radomskaya [2], Dmitry V. Bulgin [2], Renad Z. Sagdeev [1] and Yuri P. Tsentalovich [1,*]

[1] Laboratory of Proteomics and Metabolomics, International Tomography Center SB RAS, Institutskaya 3a, Novosibirsk, 630090, Russia;

[2] Research Institute of Medical Primatology, Mira Str. 177, s. Vesyoloe, Sochi, 354376, Russia;

[*] Correspondence: yura@tomo.nsc.ru; Tel.: +7-383-330-31-36; Fax: +7-383-333-13-99

Table of contents:

Figure S1. Representative ^{1}H NMR spectrum of the metabolomic extract from *M. fascicularis* serum.

Figure S2. Representative ^{1}H NMR spectrum of the metabolomic extract from *M. fascicularis* AH.

Figure S3. Representative ^{1}H NMR spectrum of the metabolomic extract from *M. fascicularis* VH.

Figure S4. Representative ^{1}H NMR spectrum of the metabolomic extract from *M. fascicularis* lens.

Figure S5. LC-OD profile of UV filters in the extract from *M. fascicularis* lens monitored at 360 nm.

Table S1. Metabolite concentrations in M. fascicularis serum, AH, lens, and VH.

Table S2. UV filters found in the *M. fascicularis* lens.

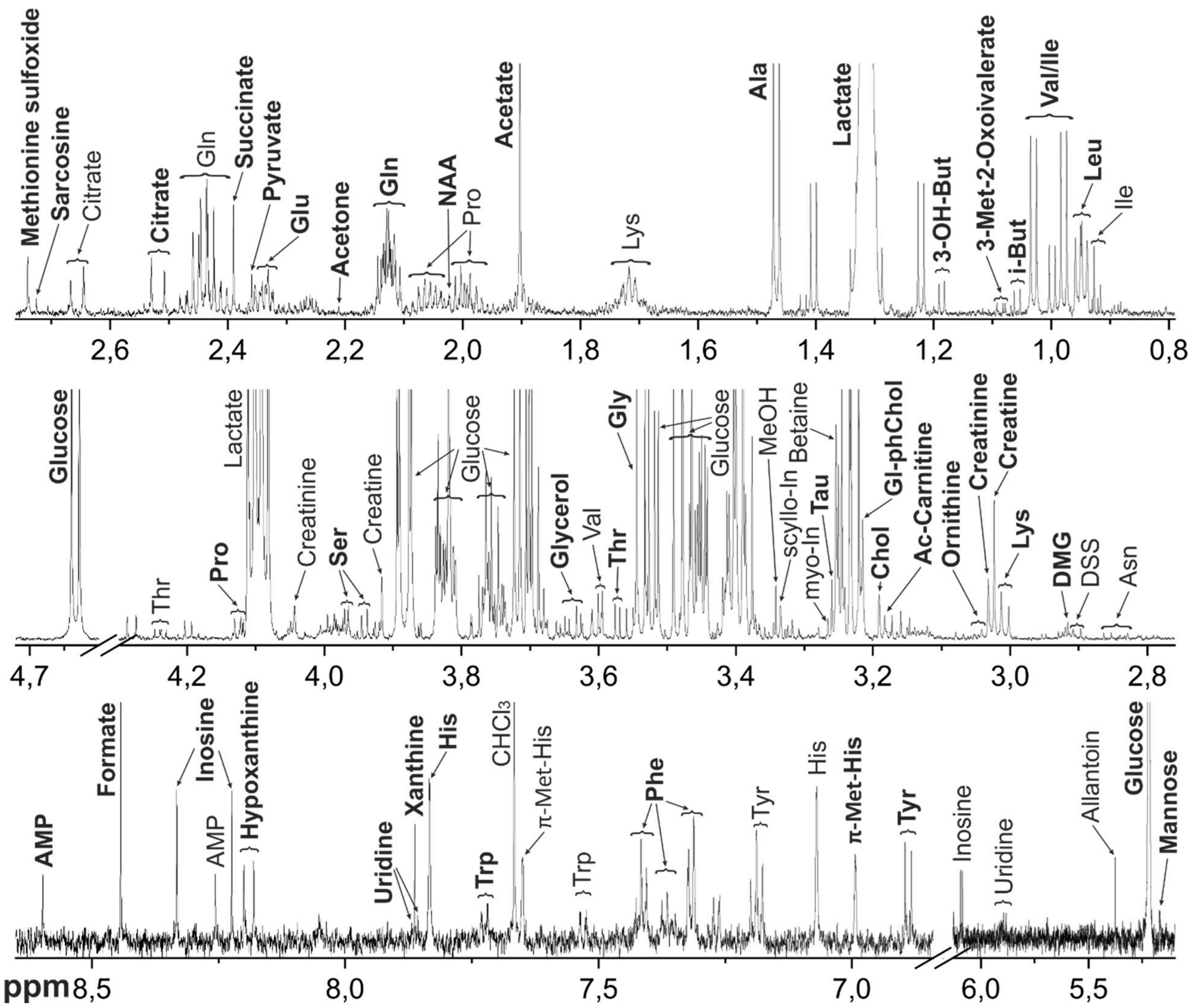

Figure S1. Representative ^{1}H NMR spectrum of the metabolomic extract from *M. fascicularis* serum. Bold letters indicate the signals used for the metabolite quantification. Abbreviations: 3-OH-But - 3-Hydroxybutyrate; Chol – Choline; DMG – Dimethylglycine; Gl-ph-Chol – Glycerophosphocholine; myo-In - *myo*-Inositol; Tau – Taurine; i-But – Isobutyrate; NAA - N-Acetylaspartate; scyllo-In - *scyllo*-Inositol; Tau – Taurine; π-Met-His - 3-Methylhistidine; τ-Met-His - 1-Methylhistidine. For amino acids and nucleotides, standard 3-letter symbols are used.

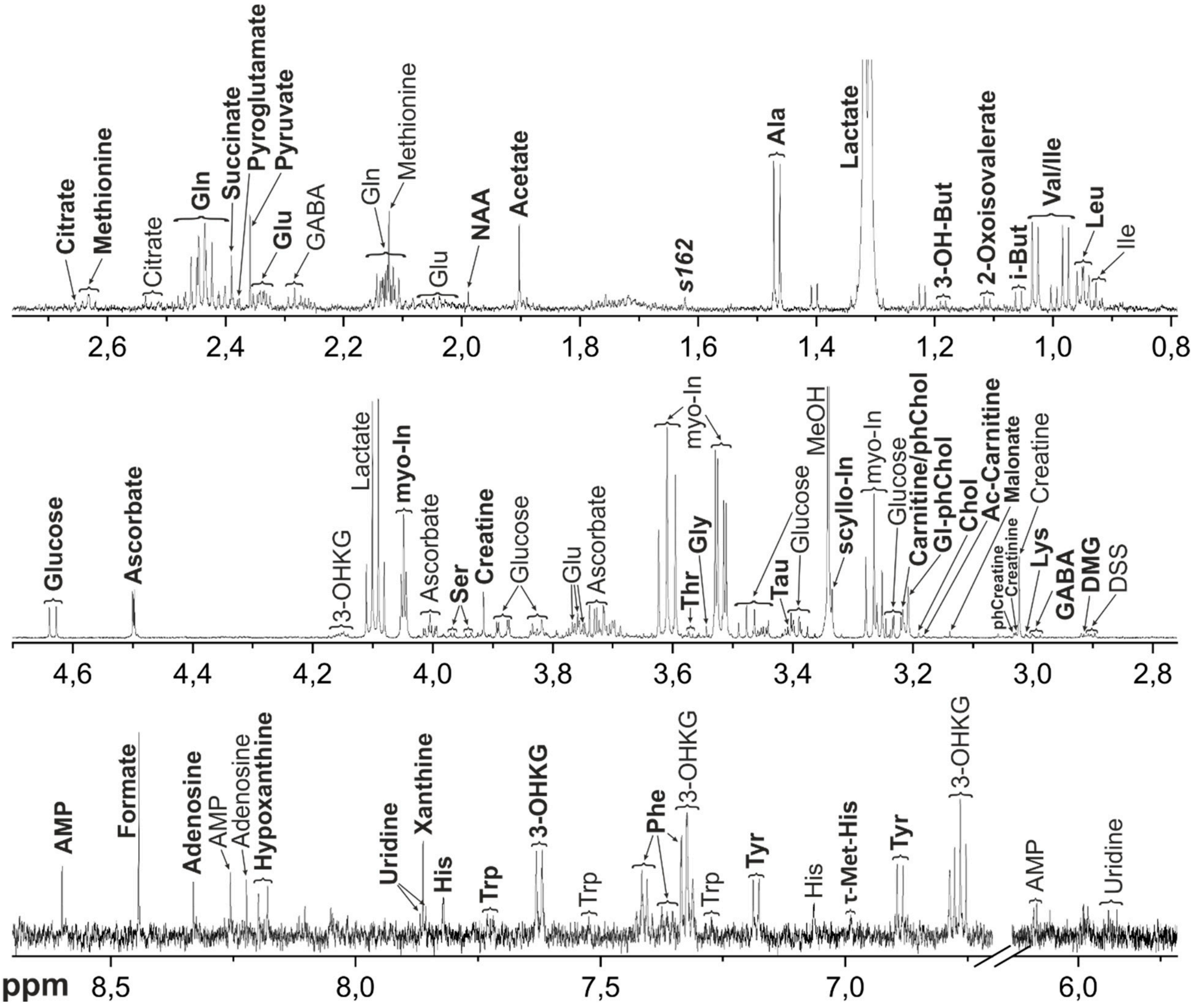

Figure S2. Representative ^{1}H NMR spectrum of the metabolomic extract from *M. fascicularis* AH. Bold letters indicate the signals used for the metabolite quantification. Abbreviations: 3-OH-But - 3-Hydroxybutyrate; Chol – Choline; DMG – Dimethylglycine; Gl-ph-Chol – Glycerophosphocholine; myo-In - *myo*-Inositol; Tau – Taurine; i-But – Isobutyrate; NAA - N-Acetylaspartate; phChol - Phosphocholine; phCreatine - Creatine phosphate; scyllo-In - *scyllo*-Inositol; Tau – Taurine; π-Met-His - 3-Methylhistidine; τ-Met-His - 1-Methylhistidine. For amino acids and nucleotides, standard 3-letter symbols are used.

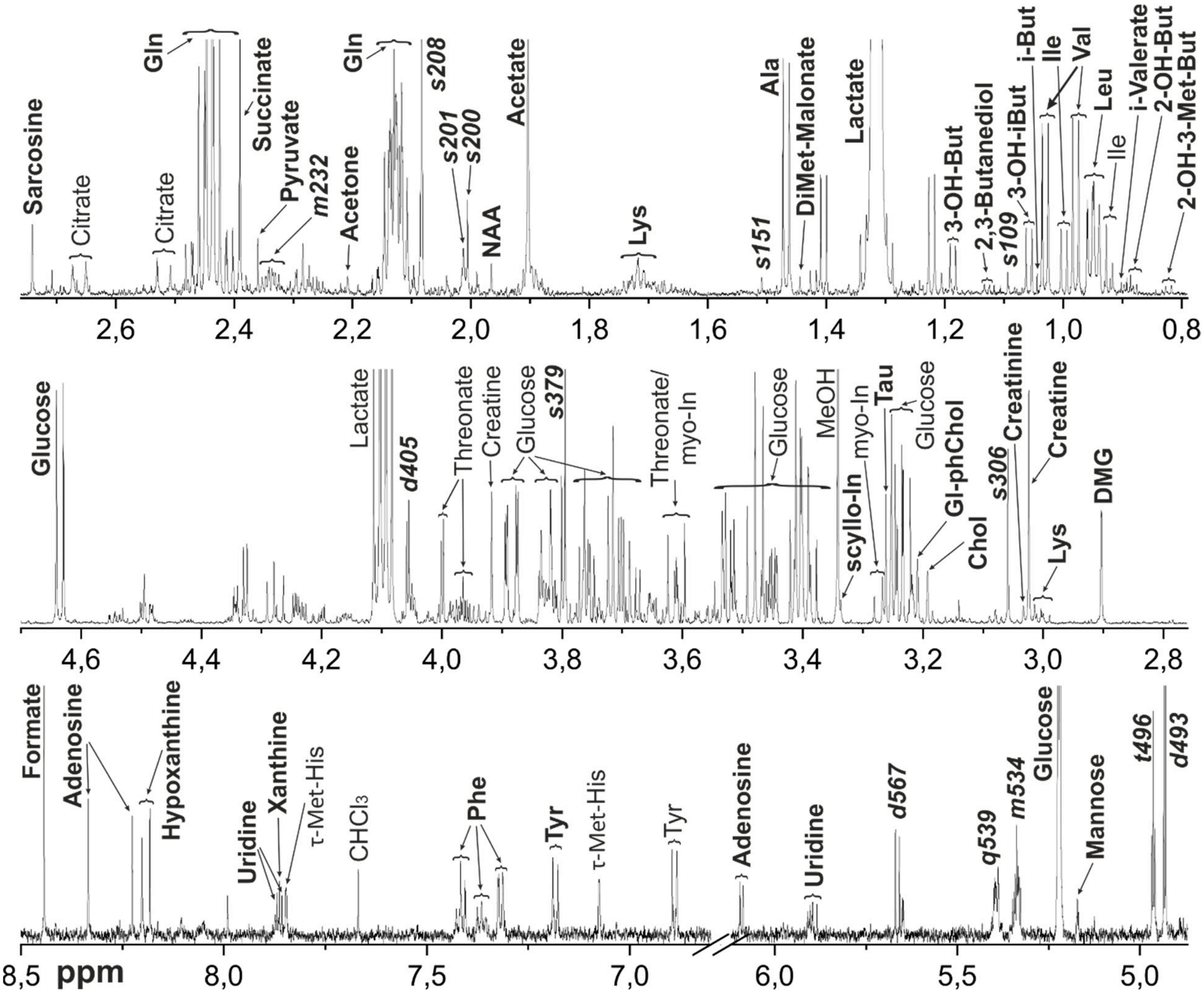

Figure S3. Representative ^{1}H NMR spectrum of the metabolomic extract from *M. fascicularis* VH. Bold letters indicate the signals used for the metabolite quantification. Abbreviations: 2-OH-3-Met-But - 2-Hydroxy-3-methylbutyrate; 2-OH-But - 2-Hydroxybutyrate; 3-OH-But - 3-Hydroxybutyrate; 3-OH-iBut - 3-Hydroxyisobutyrate; Chol – Choline; DMG – Dimethylglycine; DiMet-malonate – Dimethylmalonate; Gl-ph-Chol – Glycerophosphocholine; myo-In - *myo*-Inositol; Tau – Taurine; i-But – Isobutyrate; NAA - N-Acetylaspartate; phChol - Phosphocholine; scyllo-In - *scyllo*-Inositol; Tau – Taurine; π-Met-His - 3-Methylhistidine; τ-Met-His - 1-Methylhistidine. For amino acids and nucleotides, standard 3-letter symbols are used.

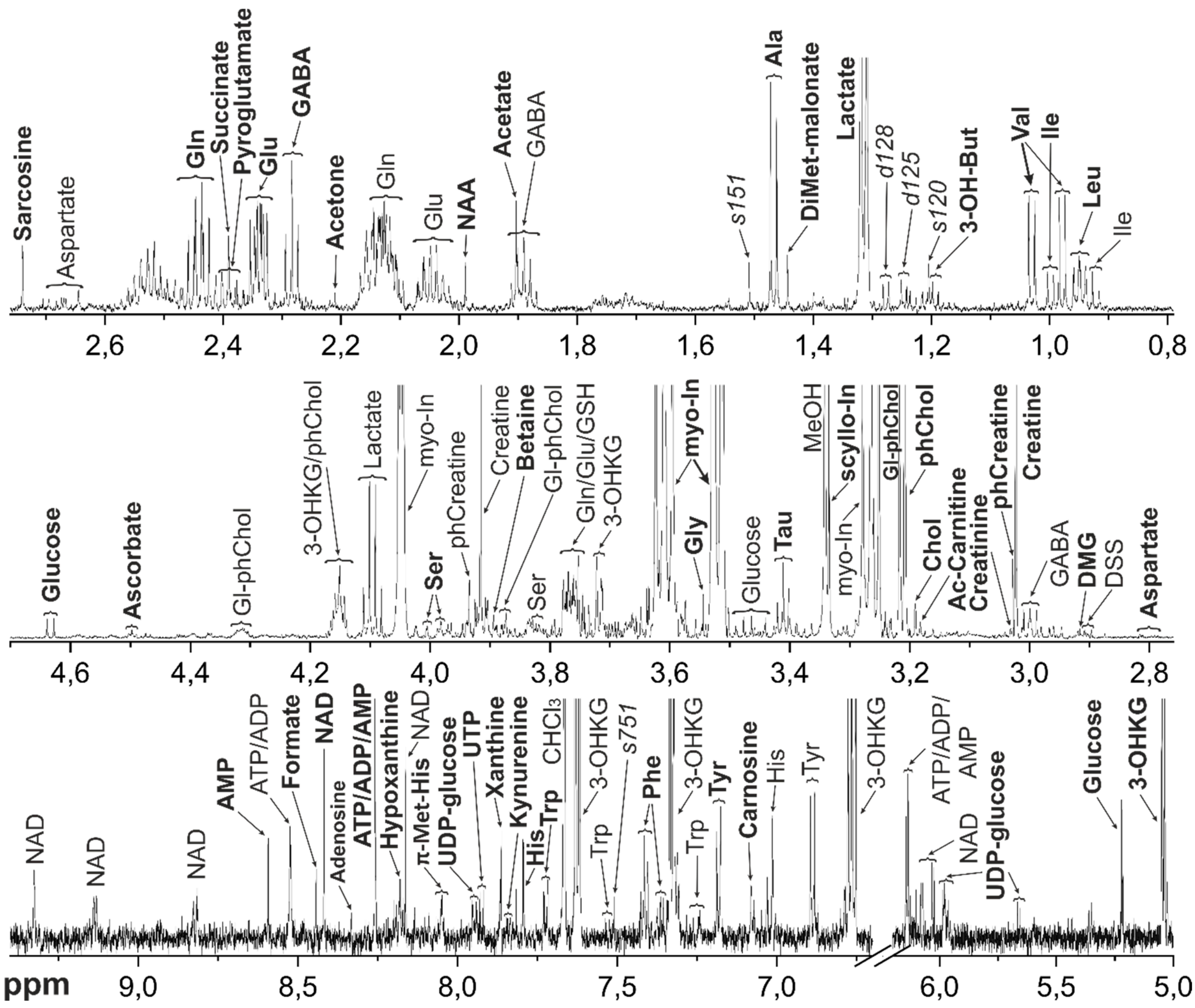

Figure S4. Representative ^{1}H NMR spectrum of the metabolomic extract from *M. fascicularis* lens. Bold letters indicate the signals used for the metabolite quantification. Abbreviations: 3-OH-But - 3-Hydroxybutyrate; Chol – Choline; DMG – Dimethylglycine; DiMet-malonate – Dimethylmalonate; Gl-ph-Chol – Glycerophosphocholine; myo-In - *myo*-Inositol; Tau – Taurine; i-But – Isobutyrate; NAA - N-Acetylaspartate; phChol - Phosphocholine; scyllo-In - *scyllo*-Inositol; Tau – Taurine; π-Met-His – 3-Methylhistidine; τ-Met-His – 1-Methylhistidine. For amino acids and nucleotides, standard 3-letter symbols are used.

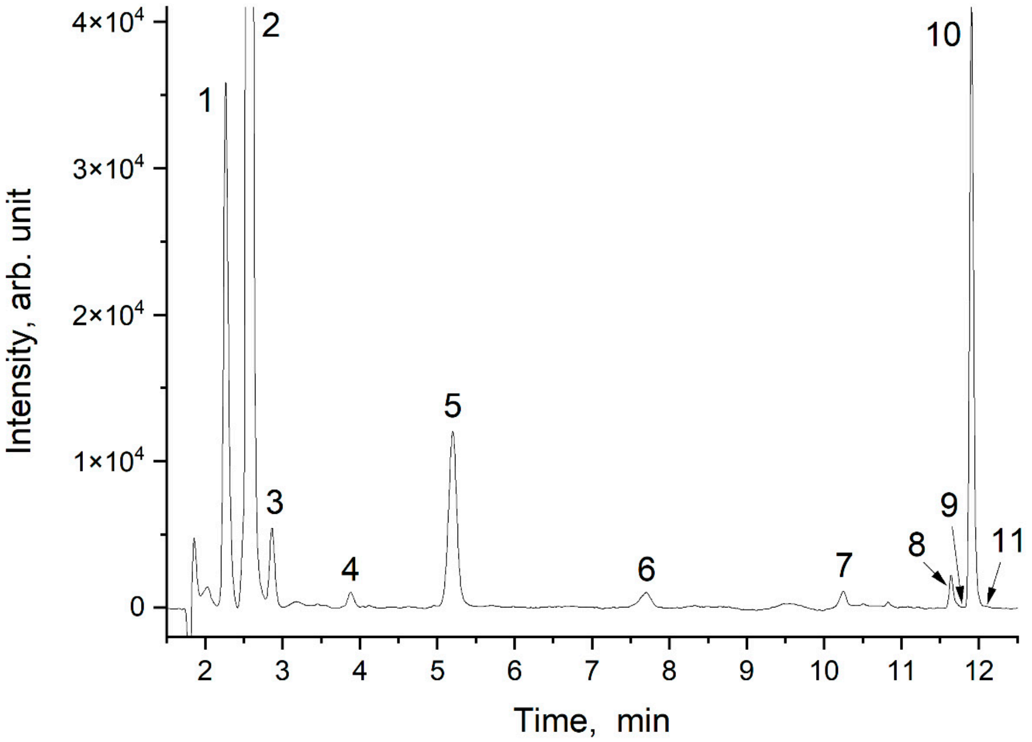

Figure S5. LC-OD profile of UV filters in the extract from M. fascicularis lens monitored at 360 nm. The signal attribution is given in Table S2.

Table S1. Metabolite concentrations in *M. fascicularis* serum, AH, lens, and VH. Values are presented as mean ± standard deviation (SD).

Metabolite	Serum *in vivo*, µM	Serum post-mortem, µM	AH post-mortem, µM	Lens post-mortem, nmol/g	VH post-mortem, µM
**Proteinogenic amino acids**					
Alanine	200 ± 30	310 ± 120	320 ± 50	600 ± 60	92 ± 29
Asparagine	88 ± 29	53 ± 10	30 ± 12	ND	ND
Aspartate	ND[1]	ND	ND	119 ± 18	ND
Glutamate	135 ± 17	190 ± 30	180 ± 40	1700 ± 300	ND
Glutamine	600 ± 40	640 ± 120	870 ± 70	1570 ± 170	750 ± 210
Glycine	340 ± 40	400 ± 50	63 ± 11	133 ± 16	18.0 ± 2.5
Histidine	85 ± 19	101 ± 18	57 ± 8	150 ± 50	20 ± 8
Isoleucine	70 ± 12	64 ± 11	56 ± 7	109 ± 16	29 ± 7
Leucine	119 ± 20	104 ± 13	132 ± 13	200 ± 40	71 ± 15
Lysine	148 ± 20	196 ± 26	81 ± 10	N/A	60 ± 15
Methionine	N/A[2]	N/A	55 ± 8	ND	ND
Phenylalanine	57 ± 12	66 ± 19	82 ± 13	160 ± 50	26 ± 7
Proline	130 ± 30	170 ± 30	130 ± 30	ND	53 ± 14
Serine	56 ± 4	65 ± 16	220 ± 30	ND	ND
Threonine	100 ± 16	102 ± 14	71 ± 11	N/A	N/A
Tryptophan	36 ± 11	26 ± 6	47 ± 20	27 ± 12	ND
Tyrosine	94 ± 25	76 ± 17	62 ± 7	135 ± 26	24 ± 5
Valine	180 ± 26	166 ± 25	187 ± 16	310 ± 50	86 ± 16
**Other amino acids**					
Betaine	54 ± 13	93 ± 29	N/A	N/A	N/A
Carnitine	N/A	N/A	25 ± 13	67 ± 9	60 ± 30
Carnosine	ND	ND	ND	101 ± 27	ND
Creatine	86 ± 23	100 ± 60	280 ± 60	2300 ± 300	140 ± 60
Creatine phosphate	7.2 ± 1.6	10 ± 5	23.6 ± 2.5	180 ± 40	N/A
Dimethylglycine	24 ± 6	22 ± 5	6.4 ± 1.6	62 ± 10	ND
Ketoleucine	10 ± 5	8 ± 7	9.0 ± 1.8	N/A	ND
Methionine sulfoxide	23.6 ± 2.7	25 ± 4	ND	96 ± 23	18 ± 5
N-Acetylaspartate	22 ± 8	25 ± 10	14.4 ± 2.3	ND	ND
N-Acetylcarnitine	19 ± 7	13 ± 5	21.8 ± 2.2	89 ± 12	10 ± 4
Ornithine	40 ± 12	59 ± 21	ND	N/A	N/A
Taurine	109 ± 21	246 ± 25	108 ± 24	570 ± 160	190 ± 190
3-Methylhistidine	58 ± 12	47 ± 17	54 ± 13	60 ± 60	ND
1-Methylhistidine	ND	ND	ND	60 ± 14	ND
**Organic acids**					
2-Hydroxy-3-methylbutyrate	ND	ND	ND	ND	4.0 ± 1.2
2-Hydroxy-3-methylvalerate	ND	ND	ND	ND	3.6 ± 1.3

2-Hydroxybutyrate	14 ± 7	15 ± 6	12 ± 6	ND	9.1 ± 2.7
2-Ketoisovalerate	5.0 ± 1.6	3 ± 3	7.0 ± 1.9	ND	ND
3-Hydroxybutyrate	70 ± 60	51 ± 20	27 ± 9	60 ± 20	23 ± 7
3-Hydroxyisobutyrate	5.4 ± 2.2	10 ± 6	ND	ND	33 ± 5
3-Methyl-2-oxovalerate	13 ± 5	12 ± 8	10 ± 3	ND	ND
Acetate	340 ± 70	330 ± 100	89 ± 24	86 ± 18	270 ± 15
Citrate	86 ± 16	120 ± 30	45 ± 6	90 ± 40	46 ± 5
Dimethylmalonate	ND	ND	ND	67 ± 20	3.9 ± 0.6
Formate	67 ± 19	64 ± 9	87 ± 9	30 ± 9	58 ± 4
Fumarate	1.8 ± 0.9	5 ± 4	ND	ND	1.8 ± 0.6
GABA	ND	ND	70 ± 40	930 ± 230	ND
Hippurate	23 ± 10	9 ± 7	ND	ND	ND
Isobutyrate	7.9 ± 2.6	7.9 ± 2.4	16.4 ± 2.2	ND	5.2 ± 1.1
Isovalerate	1.9 ± 1.0	6 ± 6	ND	ND	3.4 ± 0.9
Lactate	13000 ± 5000	20000 ± 12000	12000 ± 1700	4400 ± 700	8400 ± 2200
Malonate	ND	ND	30 ± 14	33 ± 4	ND
Pyroglutamate	ND	ND	121 ± 15	340 ± 60	ND
Pyruvate	56 ± 17	21 ± 27	58 ± 14	17 ± 5	15 ± 5
Sarcosine	6.1 ± 1.4	6.1 ± 2.4	5.0 ± 1.8	ND	ND
Succinate	22 ± 6	40 ± 40	46 ± 7	39 ± 24	49 ± 14
Threonate	ND	ND	ND	ND	468 ± 24
**Antioxidants**					
Ascorbate	ND	ND	590 ± 150	63 ± 25	83 ± 9
Ergothioneine	0.7 ± 1.0	3.0 ± 2.9	46 ± 21	140 ± 60	ND
GSH	ND	ND	ND	83 ± 22	ND
GSSG	ND	ND	ND	320 ± 70	ND
**Molecular UV filters**					
3-OHKG	ND	ND	160 ± 60	2200 ± 700	ND
3-OHCKAG[3]	ND	ND	ND	1.8 ± 0.9	ND
3-OHKDG[3]	ND	ND	ND	0.7 ± 0.4	ND
3-OHKN[3]	ND	ND	ND	4.9 ± 1.1	ND
AHBDG[3]	ND	ND	ND	5.8± 2.8	ND
AHBG[3]	ND	ND	ND	109 ± 18	ND
KN	ND	ND	ND	80 ± 50	ND
Me-3-OHKG[3]	ND	ND	ND	9.4 ± 1.1	ND
**Alcohols, ketones, amines, and carbohydrates**					
Acetamide	ND	ND	ND	44 ± 13	ND
Acetone	5 ± 7	3.3 ± 1.7	1.6 ± 1.0	8.0 ± 2.3	ND
Choline	9.5 ± 1.5	19 ± 4	17 ± 4	48 ± 10	22 ± 4
Dimethylamine	ND	1.1 ± 0.6	ND	6.2 ± 1.0	ND
Glucose	4000 ± 800	5100 ± 1300	1100 ± 300	430 ± 130	1900 ± 300
Glycerol	130 ± 50	140 ± 60	60 ± 14	N/A	66 ± 26

Glycerophosphocholine	66 ± 5	57 ± 11	70 ± 40	320 ± 100	14 ± 12
Mannose	41 ± 7	24 ± 7	ND	ND	22 ± 5
myo-Inositol	92 ± 11	110 ± 40	2900 ± 1100	40000 ± 5000	180 ± 40
Phosphocholine	ND	ND	22 ± 8	880 ± 120	ND
Phosphoethanolamine	ND	ND	ND	180 ± 40	ND
scyllo-Inositol	21.4 ± 1.2	21 ± 6	90 ± 50	690 ± 210	13 ± 4
**Nitrogenous bases, nucleotides, nucleosides**					
ADP	ND	ND	ND	70 ± 50	ND
Allantoin	25 ± 6	23 ± 4	ND	ND	ND
AMP	1.5 ± 2.0	4 ± 7	23 ± 11	85 ± 16	ND
ATP	ND	ND	ND	160 ± 110	ND
Creatinine	63 ± 11	80 ± 18	29 ± 8	48 ± 13	23 ± 4
Hypoxanthine	8 ± 5	28 ± 13	45 ± 8	90 ± 40	19 ± 8
Imidazole	ND	4.8 ± 2.7	ND	ND	ND
Inosinate	ND	5 ± 6	ND	9 ± 9	ND
Inosine	ND	38 ± 20	32 ± 6	15 ± 6	14 ± 6
NAD	ND	ND	ND	120 ± 60	ND
UDP	ND	ND	ND	16 ± 10	ND
UDP-glucose	ND	ND	ND	22 ± 12	ND
Uridine	7 ± 4	12 ± 4	37 ± 10	ND	13 ± 3
UTP	ND	ND	ND	110 ± 50	ND
Xanthine	6.5 ± 1.2	17.3 ± 1.3	42 ± 9	70 ± 30	9.9 ± 1.1

[1] ND – not detected. The value is below the NMR limit of detection (LOD)

[2] N/A – concentration value is not available due to NMR peak overlapping

[3] Concentration value is determined by LC-OD method

Table S2. UV filters found in the *M. fascicularis* lens.

Peak number	UV filter	Chemical formula	Retention time, min	m/z	λ_{max}, nm
1	unknown	N/A	2.3	N/A	368
2	3-OHKG	$C_{16}H_{22}N_2O_9$	2.6	387.140	367
3	3-OHKN	$C_{10}H_{12}N_2O_4$	2.9	225.087	371
4	3-OHKDG	$C_{22}H_{32}N_2O_{14}$	3.9	549.193	378
5	KN	$C_{10}H_{12}N_2O_3$	5.2	209.092	362
6	Me-3-OHKG	$C_{17}H_{24}N_2O_9$	7.7	401.155	371
7	unknown	N/A	10.3	445.141	369
8	unknown	N/A	11.6	445.141	368
9	3-OHCKAG	$C_{16}H_{19}NO_9$	11.7	370.113	407
10	AHBG	$C_{16}H_{19}NO_9$	11.9	372.129	363
11	AHBDG	$C_{22}H_{31}NO_{14}$	12	534.182	365
